# Impact of Conditional miRNA126 Overexpression on Apoptosis-Resistant Endothelial Cell Production

**DOI:** 10.1371/journal.pone.0126661

**Published:** 2015-05-11

**Authors:** Bo Liu, YiGang Li

**Affiliations:** Department of Cardiology, Xinhua Hospital Affiliated to Shanghai Jiaotong University School of Medicine, Shanghai, China; Bristol Heart Institute, University of Bristol, UNITED KINGDOM

## Abstract

The activation of endothelial cells is essential to repair damage caused by atherosclerosis via endothelial cell proliferation and migration. Overexpression of VEGF (vascular endothelial growth factor) and the downstream gene, B-cell lymphoma-2 (BCL-2) could result in apoptosis-resistant endothelial cells, which are responsible for aggravated hyperplasia and instable plaques generation. Previous studies have shown that miRNA126 could regulate the expression of VEGF. Here, we verified the existence of a miRNA126 binding site in VEGF’s 3’UTR. Additionally, VEGF regulated BCL-2 expression via AP1 (Activator Protein 1) binding site in BCL-2’s promoter. Next, we established an apoptosis-resistant endothelial cell line and constructed a lentiviral vector to express miRNA126 under the control of the BCL-2 promoter to investigate whether conditional expression of miRNA126 could modulate VEGF and BCL-2 expression in apoptosis-resistant endothelial cells. This lentiviral system specifically expressed miRNA126 in cells with high BCL-2 levels, downregulated VEGF expression, inhibited MAPK pathway activation and downregulated BCL-2 expression via suppression of AP1, and as a whole, reduced apoptosis-resistant endothelial cells, while the effects of miRNA126 on normal endothelial cells were relatively small. Our results demonstrate that conditional miRNA126 overexpression under the control of the downstream BCL-2 promoter provides a flexible regulatory strategy for reducing the apoptosis-resistant endothelial cells without having a significant impact on normal endothelial cells.

## Introduction

Atherosclerosis, the most common vascular disease caused by arterial sclerosis, develops from an accumulation of lipids and complex carbohydrates on vascular walls, could result in hemorrhaging, thrombogenesis, proliferation of fibrous tissue, calcium deposition, and the gradual decay and calcification of the atrial wall medial layer[1]. Previous studies have shown that the activation of endothelial cells plays an important role in the development of atherosclerosis. Activated endothelial system and up-regulated inflammatory cytokines, adhesion proteins and chemokines are often observed on endothelial cells exposed to risk factors [2]. The inflammatory, high lipid environment could also injure vascular endothelial cells, especially in plaque-containing areas [3,4]. According to vascular endothelial injury and repair theory, new endothelial cells, mainly originating from proliferating vessel endothelial cells and from blood endothelial progenitor cells at the plaque lesions, could fill in the damaged regions to resist apoptosis of endothelial cells [5]. These endothelial cells tend to proliferate at extraordinary rates [6]. It is reported that, during the repair of a vascular injury, endothelial cells express and secrete high levels of VEGF and BCL-2, which could accelerate the differentiation of endothelial progenitor cells into endothelial cells [7]. However, these endothelial cells lost the ability to repair themselves through spontaneous apoptosis and proliferation under normal conditions and are resistant to apoptosis, forming so called apoptosis-resistant endothelial cells, which are responsible for aggravated hyperplasia and instable plaques generation[8]. Thus, Selective inhibition of apoptosis-resistant endothelial cells may be a favorable strategy for treating atherosclerosis, while non-selective inhibition on endothelial cells may directly or indirectly increase the shedding of the vascular endothelial cells and aggravate atherosclerosis [9,10].

Based on the endothelial injury and repair mechanism, selective inhibition of BCL-2, the key-regulating gene for apoptosis-resistant endothelial cells, might be of therapy value for atherosclerosis. It is reported that VEGF can regulate the expression of BCL-2 in endothelial cells [11] and VEGF expression could be regulated by miRNA126 in various tissues [12, 13, 14]. miRNA126 might thus be a suitable candidate for regulating the expression of BCL-2 and VEGF in endothelial cells and overexpression miRNA126 might be able to reduce apoptosis-resistant endothelial cells via downregulating BCL-2 and VEGF. This study is therefore aimed to demonstrate the role of BCL-2 in the production of apoptosis-resistant endothelial cells and to observe the effects of overexpressing miRNA126 on apoptosis-resistant endothelial cells and BCL-2/VEGF expression.

## Materials and Methods

### Establishment and Validation of Apoptosis-resistant Rat Aortic Endothelial Cells

Rat aortic endothelial cells (RAECs, Cell Bank of China Academy of Science) were stimulated by oxidized low-density lipoprotein (OX-LDL, Sigma, Missouri, USA) to induce apoptosis-resistant endothelial cells (ARAECs). Briefly, RAECs were cultured in ECM medium (ScienCell, CA, USA) containing 10% fetal bovine serum (Invitrogen, CA, USA). Cells in log-phase growth were resuspended and stained with Trypan blue for vital counting. The cells were seeded into 6-well plates at 2 × 10^5^ cells/well. OX-LDL was added and the final concentration of OX-LDL was increased gradually (1 to 2, 2 to 5, 5 to 10, 10 to 20, 20 to 50, and 50 to 100μg/mL, increased every three days) along with passage or medium replacement. The cells obtained were renamed ARAECs.

RAECs and ARAECs were seeded into 6-well plates at 2 × 10^5^ cells/well, treated 24 hour later with 50 μg/mL OX-LDL for 24 hours and then stained with Annexin V: FITC Apoptosis Detection Kit II (BD, New Jersey, USA). The cells were resuspended by trypsinization, washed with dPBS and resuspended in 500 μL binding buffer with 5μL Annexin V-FITC in the dark for 10 minutes. The cells were then stained with 5 μL Propidium Iodide for 5 minutes. Apoptosis was analyzed on BD-FACS Calibur using the FITC (FL1) channel and the PI (FL2) channel at an excitation wavelength of 488 nm.

For the cell viability assay, RAECs and ARAECs were seeded into 96-well plates, cultured under normal conditions, and at different time points were treated with 10 μL CCK-8 solution (Dojindo, Tokyo, Japan) for 4 hours. The absorbance at 450 nm was determined on a plate reader.

To verify the resistance of the cells to apoptosis, we detected the activity of Caspase3, a marker of early apoptosis. The cells were seeded to 6-well plates, at a concentration of 2 × 10^5^ per well, and cultured under normal conditions for 24 hours. The RAECs and ARAECs treated with 50 μg/mL OX-LDL for 6 hours were collected and subjected to Caspase 3 activity assay (Roche, Mannheim, Germany) in accordance with the manual of the assay kit.

### miRNA126, VEGF and BCL-2 Expression

Total RNA samples were extracted from RAECs and ARAECs using Trizol and used to prepare cDNA with a M-MLV reverse transcription kit with the following primers: Rat U6 snRNA: 5’-ATGGAACGCTTCACGAATTTG-3’ and miRNA126 (MIMAT0000832): 5’-GTCGTATCCAGTGCGTGTCGTGGAGTCGGCAATTGCACTGGATACGACCGCAT-3’. The RNA contents were detected by the fluorescence dye method following the manufacturer’s instructions. The following primers were used to detect U6 snRNA and miRNA126: U6-forward primer: 5’-TGCCTGCTTCGGCAGCACA-3’ and U6-reverse primer: 5’-ATGGAACGCTTCACGAATTTG-3’ produced a length of 101 bp; and miRNA-126-forward primer: 5’-TCGTACCGTGAGTAATAATGCG-3’ and miRNA-26-reverse primer: 5’-GTCGTATCCAGTGCGTGTCGTG -3’. The parameters were 40 cycles of denature at 95°C for 5 s, anneal at 60°C for 20 s and extend at 72°C for 20 s. Relative contents were analyzed using the 2^ΔΔCt^ method with U6 as a reference gene. Each RNA sample was run in triplicate.

To determine the secretion of miRNA126 by the cells, we collected and measured miRNA126 in the supernatants of the two groups. RNA was extracted from 250 μL supernatant added with 750 μL Trizol, and detected as mentioned above. Moreover, VEGF in the supernatant was also determined by using Elisa kit (Invitrogen, CA, USA) in accordance with the instructions of the assay kit.

RAECs and ARAECs were rinsed with 1 ml of cold dPBS and treated with 1 ml of cell lysis buffer (50 mM pH 8.0 Tris, 1 mg/mL leupeptin, 150 mM NaCl, 0.5% Nonidet P-40, 5 mM EDTA, 100 mM PMSF, 1mM DTT, and 1 mg/mL aprotinin) for protein extraction. A BCA assay was used to determined protein concentrations. Protein samples (10μg/Lane) were separated by 10% SDS-PAGE and transferred to PVDF membranes using the wet transfer technique. Blots were blocked in TBST containing 5% nonfat milk at room temperature for 2 hours and incubated with the primary antibodies (VEGF, 1:300; BCL-2, 1:500; beta actin, 1:800) at 4°C overnight. The membranes were then rinsed with TBST and incubated with the secondary antibodies for 2 hours. ECL chemiluminescence substrate and x-ray film were used to detect the bands and the relative optical densities were analyzed with image processing software. The relative contents of VEGF or BCL-2 were equal to the optical density of target band divided by the optical density of the beta-actin band.

### Analysis of Target Relationship between miRNA126 and VEGF

TargetScan was used to predict a theoretical target of miRNA126 in the mRNA sequence of the VEGF gene (NM_001287107.1). Rat total RNA was extracted from 1×10^7^ normal endothelial cells and reverse transcribed into cDNA for amplification of VEGF’s 3’UTR. The primers (containing XbaI cutting site) used were: 5’-GACGTGATGTTAATATCT-3’ and 5’-AATCTGTGTTTCCAATCTCTCTC-3’. PCR parameters were 32 cycles of denaturation at 95°C for 10 s, annealing at 58°C for 30 s and extension at 72°C for 30 s. The PCR product was digested and inserted into the linear PGL3-Promoter (Promega, Wisconsin, USA). The obtained plasmid was mutated from 5’-ATTATTA-3 to 5’-TAATATT-3’ by introducing point mutations. The two vectors were named PGL3-WT-VEGF and PGL3-MT-VEGF. After sequencing confirmed the vectors were constructed successfully, endotoxin-free plasmids were prepared. miRNA126 mimics, inhibitors and NC were synthesized chemically (Invitrogen).

293 cells in log-phase growth were seeded into 96-well plates and transfected 24 hours later using Lipofectamine 2000(Invitrogen) following to the manufacturer’s instructions. Cells were also transfected with pGL-TK (Promega, 50 ng/well) as a luciferase reference. Luciferase activities were measured by the dual luciferase reporter assay system (Promega) 48 hours after transfection.

### Analysis on miRNA126 and BCL-2 Regulation

A pshRNA-miRNA126-inhibitor was designed according to the mature sequence of miRNA126. The sense (5’-GATCCTCGTACCGTGAGTAATAATGCGCTTCCTGTCAGACGCATTATTACTCACGGTACGATTTTTG-3’) and antisense shRNA sequences were synthesized(Invitrogen). BamHI and EcoRI cut sites were added to both ends and the oligonucleotides were annealed and cloned into pshRNA-H1 (SBI, CA, USA). SiRNA design software was used to design pshRNA-VEGF, pshRNA-BCL-2 and pshRNA-AP1 to target VEGF, BCL-2 and AP1, respectively. The corresponding hairpin templates were designed (Table 1), synthesized, annealed and cloned into pshRNA-H1.

**Table 1:** Corresponding hairpin templates for siRNA target sites. Portions of the target sequences are underlined.

Gene	Sequence
VEGF	5’-GATCC*GAGTACCCCGATGAGATAG*CTTCCTGTCAGACTATCTCATCGGGGTACTCTTTTTG-3’
BCL-2	5’-GATCC*GCAGAGATGTCCAGTCAGC*CTTCCTGTCAGACAGCTTATAATGGATGTACTTTTTG-3’
AP1	5’-GATCC*GTTCATGGAGATGCTGTCT*CTTCCTGTCAGACTGCTCGTCGGTCACGTTCTTTTG-3’

PCR primers for amplification of VEGF and BCL-2 coding sequences (Table 2) were designed and cDNA reversely transcribed from RNA was used as templates. The PCR products were digested with EcoRI and BamHI and cloned to pcDH1 (SBI) for VEGF and BCL-2 expression. The primers to amplify the BCL-2 promoter and the precursor sequence of miRNA126 are shown in Table 2. A fragment of approximately 2000 bp before the BCL-2 gene transcriptional start site was amplified and inserted into pcDNA-CMV-BCL-2, replacing the CMV promoter to produce the vector pcDNA-Pro-BCL-2. The precursor sequence of miRNA126 (a length of 467 bp) was amplified and cloned into pcDNA-Pro-BCL-2, replacing the BCL-2 gene to construct a miRNA126 expression vector under the control of BCL-2 promoter.

**Table 2:** Primers for amplification of VEGF and BCL-2 coding sequences and BCL-2 promoter and precursor sequence of miRNA126.

VEGF coding sequence	5’-CGGAATTCGCCACCATGAACTTTCTGCTCTC-3’
	5’-CGGGATCCTCACCGCCTTGGCTTGTCAC-3’
BCL-2 coding sequence	5’-CGGAATTCGCCACCATGGCGCAAGCCGGGAGA-3’
	5’- CGGGATCCTTCACTTGTGGCCCAGGTATGC-3’
BCL-2 promoter	5’- GACTAGTAGAAAGGGTCCATTGGATTGG-3’
	5’- GACTAGTTTCAAATTCCTTTCAGCT-3’
miRNA126 precursor sequence	5’- CGGAATTCGCCACCTGTCTTTCCGACCTGC -3’
	5’- CGGGATCCTGAGTCTGGAGATGCAA-3’

Following the manufacturer’s instructions, the expression or shRNA vectors and Lentivirus Package plasmid mix were co-transfected into 293T producer cells using Lipofectamine 2000 (Invitrogen). The supernatants were collected 48 hours later and cleared by centrifugation and filtering through 0.45 μm PVDF membranes (Millipore, CA, USA). The viral titer was evaluated by a gradient dilution. The packaged lentiviruses were named Lv-inhibitor-miRNA126, Lv-CMV-VEGF, Lv-CMV-BCL-2 and Lv-Pro-BCL-2.

For miRNA126 content detection, ARAECs were divided into five groups: control group, control virus group, miRNA126 inhibition group, VEGF silencing + miRNA126 inhibition group and VEGF silencing control + miRNA126 inhibition group. The ARAECs in log-phase growth were seeded into 6-well plates at 2×10^5^ cells/well and cultured under normal conditions for 24 hours. Lentiviral solutions were added at a multiplicity of infection (MOI) of 10. The cells were cultured for 72 hours and the infection efficiencies were determined by analyzing the GFP(Green Fluorescent Protein, GFP) fluorescence using a fluorescent microscope. Total RNA was extracted for mature miRNA126 detection.

ARAECs were divided into seven groups: control group, control virus group, VEGF overexpression group, VEGF+ BCL-2 (pro) group, VEGF + BCL-2 (CMV) group, VEGF + BCL-2 (pro) + AP1 silencing group, and VEGF + BCL-2 (pro) + AP1 silencing group. The infection was performed as mentioned above, except the seeding density was 1×10^5^ cells/well and MOI was adjusted to 5. VEGF and BCL-2 protein levels were detected 72 hours after infection.

### Effects of Targeted miRNA126 Expression on Apoptosis of RAECs and ARAECs and Associated Proteins

RAECs and ARAECs in log-phase growth were seeded into 6-well plates at 2×10^5^ cells/well and cultured under normal conditions for 24 hours. Lentiviral solutions were added at MOI 10 and the infection efficiencies were determined by fluorescent microscopy 72 hours later. Real time PCR was used to detect miRNA126 contents and western blotting was used to detect VEGF, AP1 and BCL-2.

### Time-course of Lv-Pro-miRNA126 Targeted Inhibition

RAECs and ARAECs in log-phase growth were infected with Lv-Pro-miRNA126 and were collected 24 hours later. The levels of miRNA126, VEGF, and BCL-2 and cell apoptosis were measured using real-time PCR, western blotting and double-stain apoptotic kit, respectively. For apoptosis analysis, the cells were divided into four groups: RAECs control, ARAECs control, ARAECs + Lv-Pro-miRNA126 and A-RAECs + Lv-shRNA-BCL-2 groups.

### Detection of Effects of Lv-Pro-miRNA126 on Inflammatory Factors in ARAECs Induced by OX-LDL and the MAPK Pathway

The cells were divided into five groups: RAECs control, ARAECs control, ARAECs + OX-LDL, ARAECs + Lv-Pro-miRNA126 + OX-LDL and ARAECs + Lv-control-OX-LDL. Cells were incubated with OX-LDL at a final concentration of 50 μg for 48 hours. After that, the total protein was extracted and the corresponding proteins were detected by western blotting.

### Statistical Analysis

SPSS13.0 was used for statistical analysis. All data are presented as the mean ± standard deviation (SD). Factorial analysis was employed for inter-group and intra-group comparison; P values<0.05 were taken as the level of significance.

## Results

### Detection of Apoptosis and Proliferation

**Fig 1:**
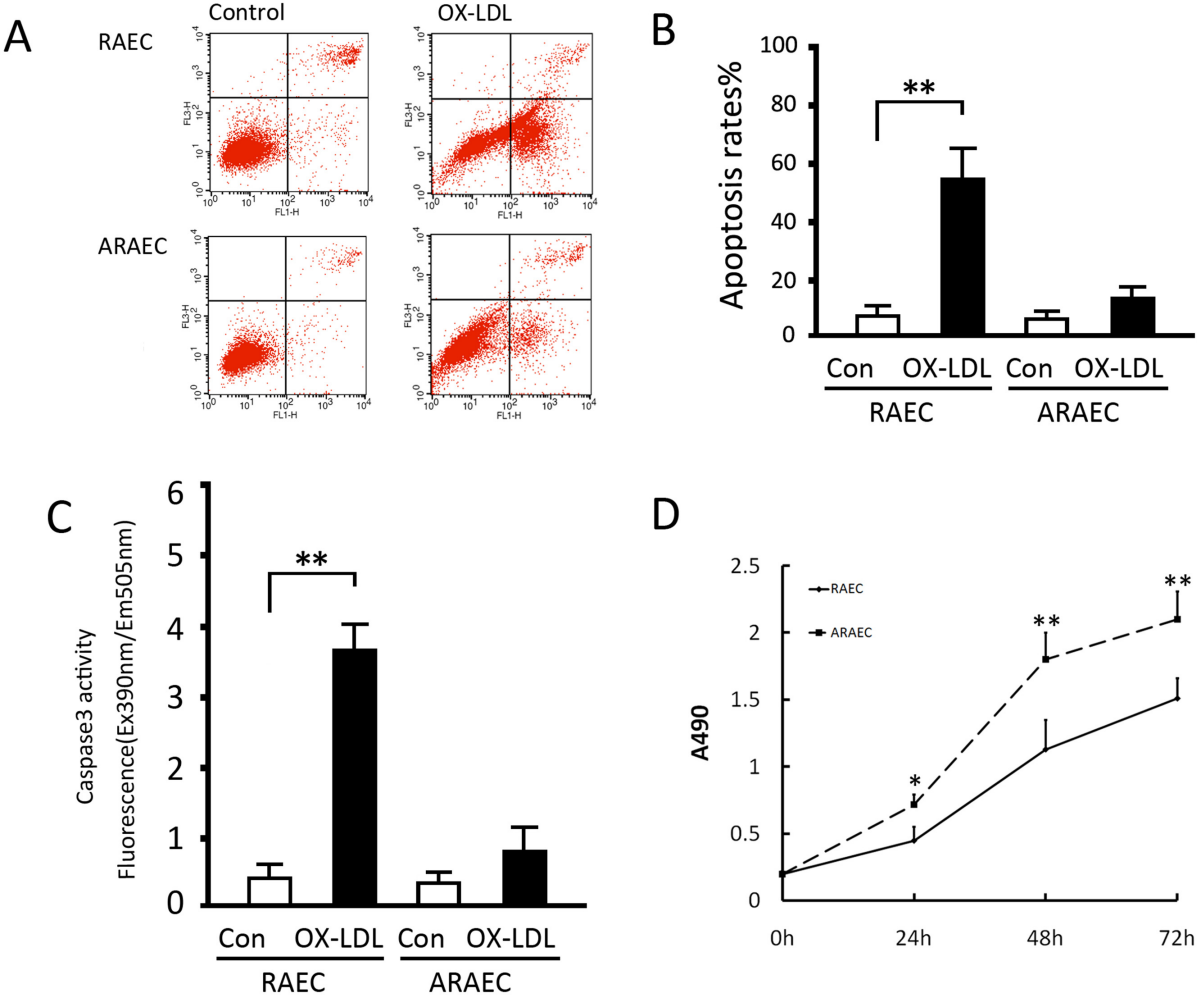
Apoptotic and proliferation profiles of RAECs and ARAECs. RAECs and ARAECs were cultured overnight and treated with or without 50 μg/mL of OX-LDL for 48 hours and then were subjected to apoptosis detection by an Annexin V:FITC Apoptosis Detection Kit II (BD). (A) Representative flow cytometry plots. (B) Quantitative measurements of flow cytometric determination of apoptotic cells. (C) Caspase3 activity assay of RAECs and ARAECs. (D) Proliferative curves of RAECs and ARAECs under normal conditions. Results are the means ± SD of at least 3 separate experiments. *, p<0.05 and **, p<0.01, when compared to the corresponding RAEC group or the indicated group.

Treatment with 50 μg/mL OX-LDL for 24 hours significantly increased the apoptotic ratio of RAECs from 7.2% to 52.4% (p<0.05). However, the apoptotic ratio of AREACs was not significantly affected by 50 μg/mL OX-LDL, only from 5.8% to 12.2% (>0.05) (Fig 1A and 1B). The results of Caspase3 activity assay indicated treatment with 50 μg/mL OX-LDL for 6 hours significantly increased caspase3 activity in RAECs in comparison with the control group (p<0.05), but not in the AREACs cells (P>0.05) (Fig 1C). Data from the cell proliferation assay showed that the proliferative activity of AREACs was higher than that of REACs at 24, 48, or 72 h under normal conditions (all p<0.05) (Fig 1D). These results verified the apoptotsis resistant characteristics and reduced proliferative activity of AREACs.

### Detection of miRNA126, VEGF and BCL-2 levels in REACs and AREACs

**Fig 2:**
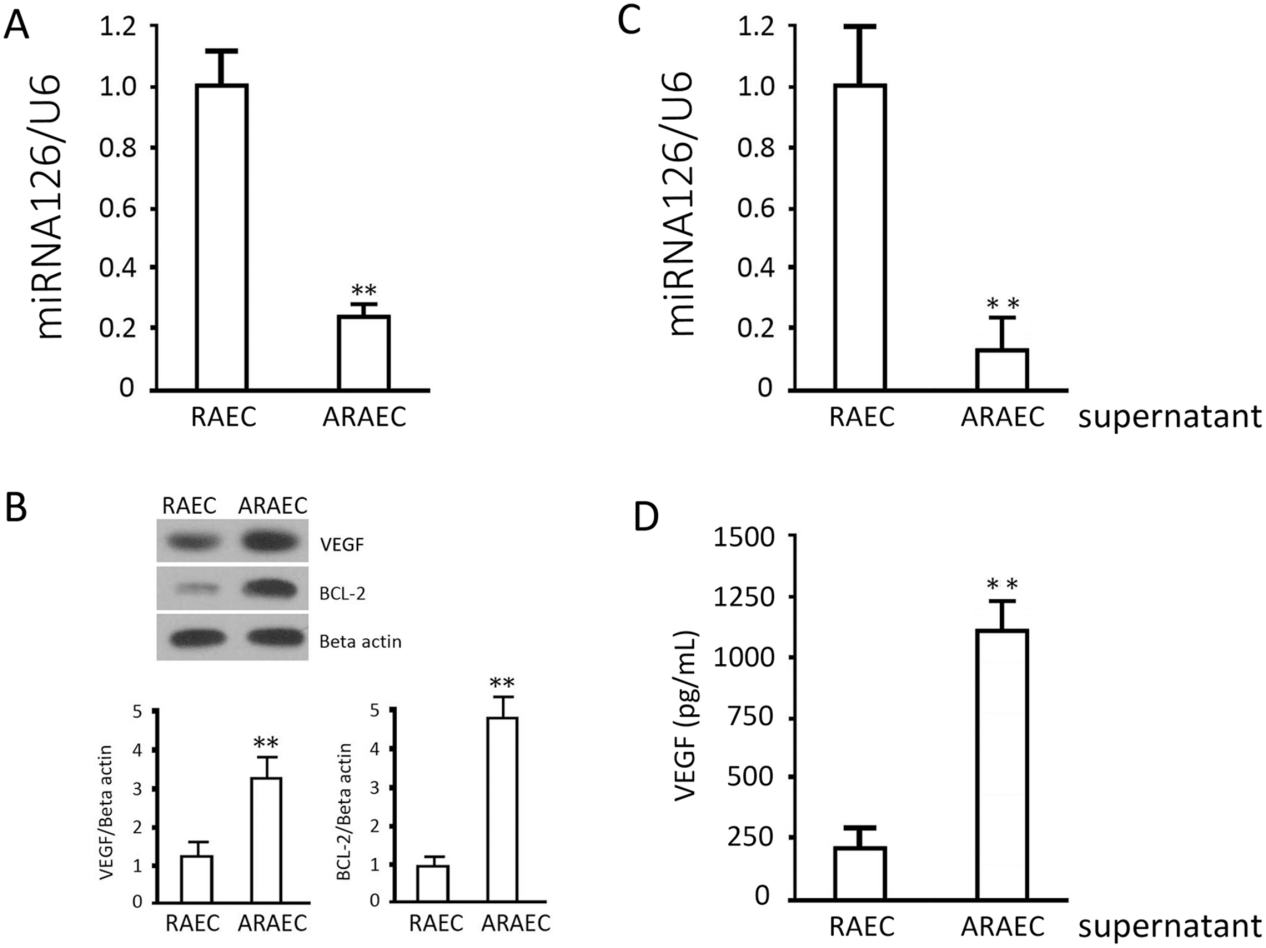
miRNA126, VEGFC and BCL-2 levels in RAECs and ARAECs. Cells were cultured under normal conditions and miRNA126, VEGFC, and BCL-2 levels were assessed with quantitative PCR and specific antibodies against VEGF and BCL-2, respectively. (A) Relative miRNA126 levels in RAECs and ARAECs. (B) Representative immunoblots of VEGFC and BCL-2 and corresponding data of optical densities. (C) Mature miRNA126 levels in the supernatants of RAECs and ARAECs. (D) VEGF concentrations in the supernatants of RAECs and ARAECs. Results are the means ± SD of at least 3 separate experiments. **, p<0.01, when compared to the corresponding RAEC group.

Real time PCR and Western blots showed significantly downregulated miRNA126 expression in AREACs (approximately 21.2% of that in REACs, p<0.05) (Fig 2A), While the protein levels of VEGF and BCL-2 were significantly higher in AREACs than those in REACS (p<0.05) (Fig 2B). The detection of miRNA126 in the supernatant demonstrated miRNA126 secretion by AREACs was significantly lower than by REACs (p<0.05) (Fig 2C), consistent with the results from detection of cellular miRNA126. The content of VEGF in the supernatant was parallel with the change in the cellular protein (Fig 2D), with higher secretion of VEGF in AREACs (p<0.05).

### Verification of a miRNA126-binding Site in VEGF’s 3’ UTR

**Fig 3:**
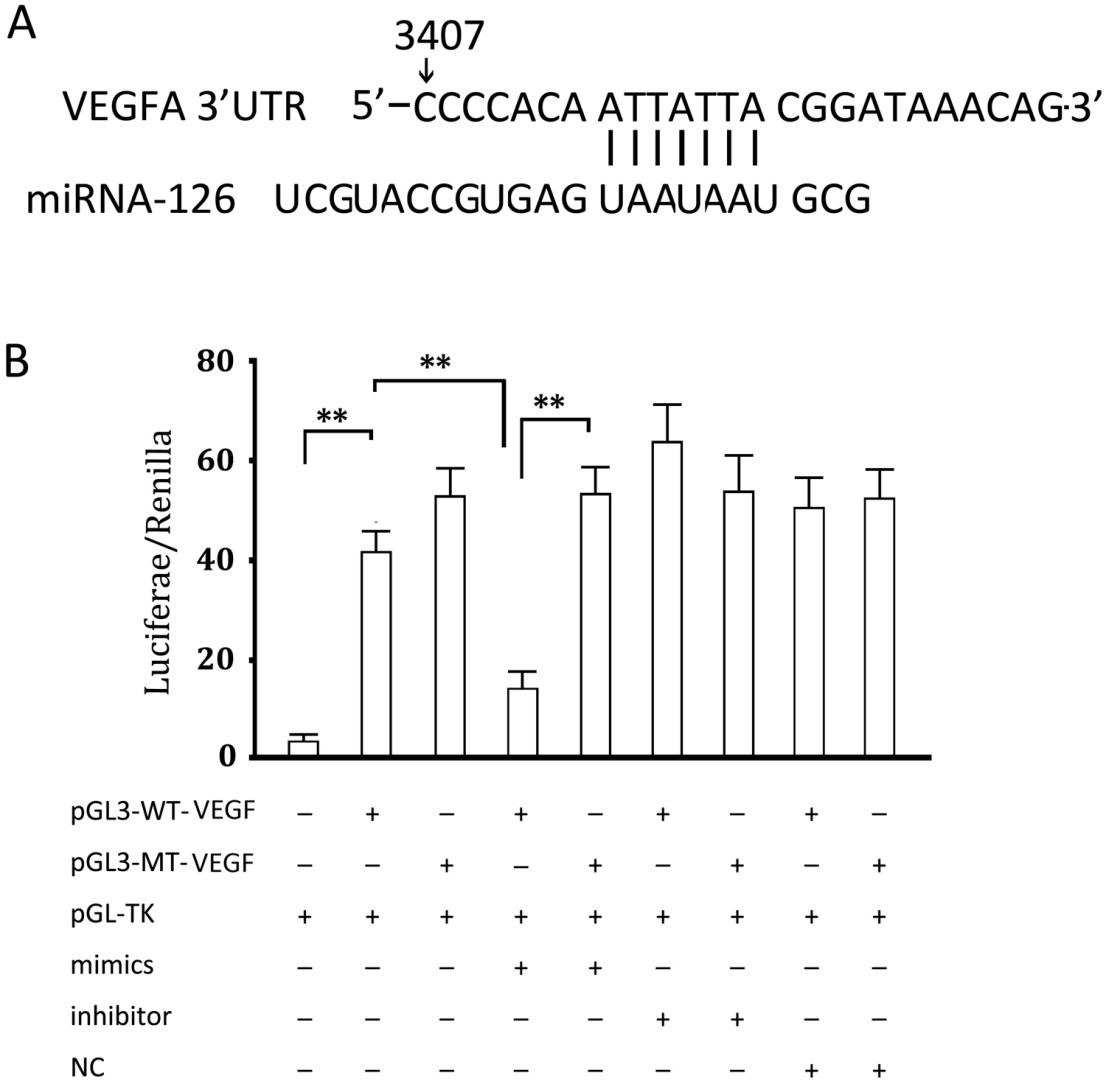
Verification of an interaction between miRNA126 and VEGFA. (a) Predicted binding site of miRNA126 in the 3’UTR of VEGFA; (b) 293 cells were transfected with pGL3-WT-VEGF or pGL3-WT-VEGF in the presence or absence of miRNA126-mimic or miRNA126-inhibitor. The histogram indicates the relative firefly luciferase activities in the different transfected groups. Error bars represent standard deviation and were obtained from at least three independent experiments. **, P < 0.01. miRNA126 regulates BCL-2 through the transcription factor AP1.

Analysis of the VEGF gene predicts a possible miRNA126-binding site. We used a luceiferase activity assay to assess whether VEGF expression is regulated by miRNA126. By comparing the co-transfected groups and groups transfected with plasmid alone, we found that a miRNA126-mimic significantly inhibited intercellular luciferase activity compared with the group transfected with the luciferase expression vector alone (p<0.05) and a miRNA126-inhibitor significantly increased the luciferase activity (Fig 3B). However, both miRNA126-mimic and miRNA126-inhibitor had no obvious effect on the luciferase activity in cells transfected with the luciferase expression vector carrying a mutated binding site (p>0.05) (Fig 3B). The cells transfected with miRNA126-NC showed a similar luciferase activity in comparison with the group transfected the luciferase expression vector alone, indicating that RNA transfection had no effect on luciferase activity. These results suggest that there is a miRNA126-binding site in the VEGF gene, as predicted.

**Fig 4:**
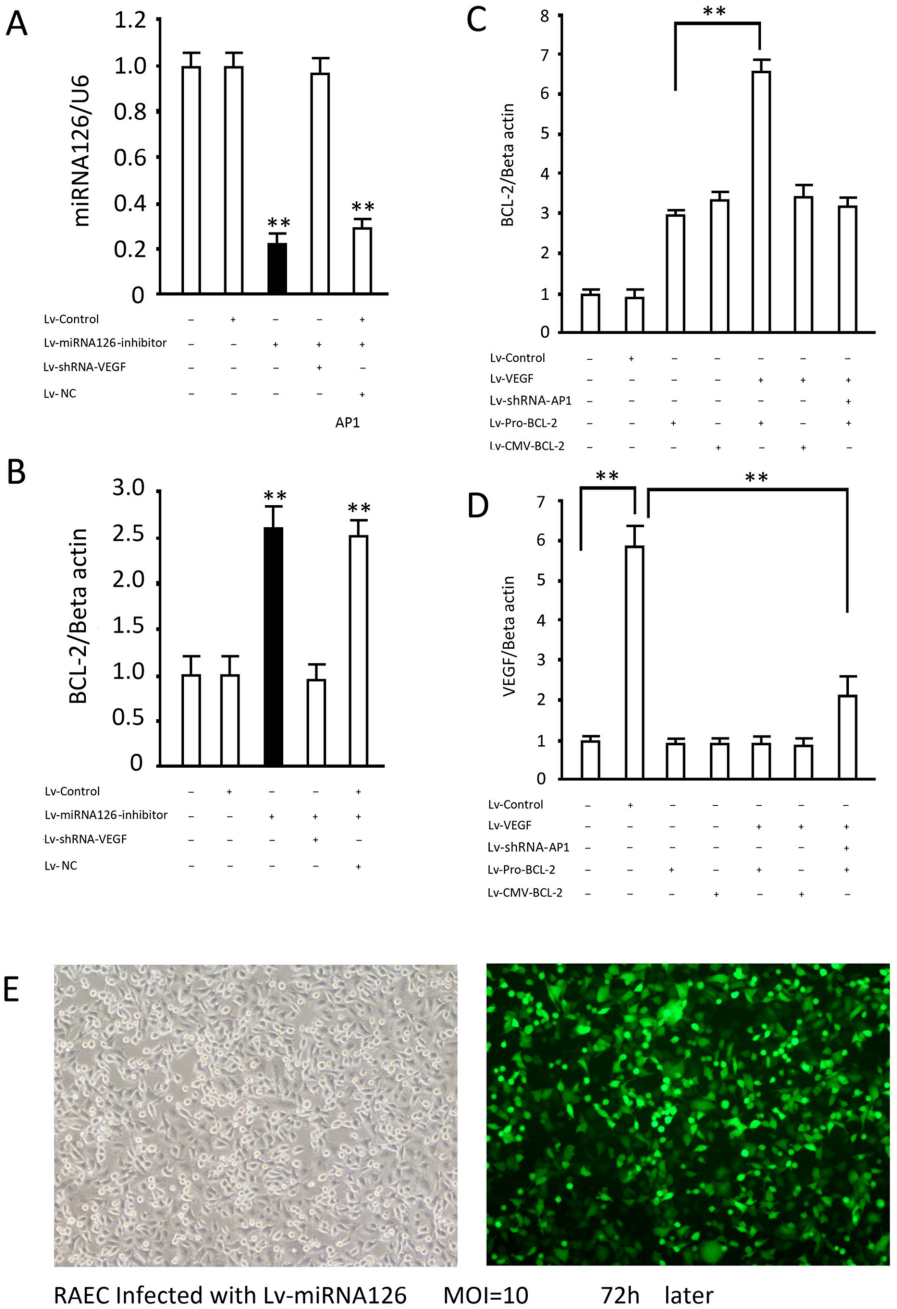
AP1 is involved in the regulation of BCL-2 by miRNA126. (A, B) RAECSs were infected with or without the indicated lentivirus and miRNA126 and BCL-2 levels were analyzed 72 hours later by quantitative PCR and western blotting, respectively. (C, D) RAECSs were infected with or without the indicated lentivirus and BCL-2 and VEGF were analyzed by western blotting 72 hours later. (E) Representative photo of GFP expression in RAECs infected with Lv-miRNA126 for gene delivery efficiency assessment 72 hours after infection. Results are the means ± SD of at least 3 separate experiments. **, p<0.01, when compared to the corresponding Lv-control group or indicated group.

The gene transduction efficiency, which was close to 100% via lentiviral experimental system in endothelial cells, lays a satisfactory foundation for successful genetic intervention (Fig 4E). In ARAECs, the antisense sequence effectively decreased endogenous mature miRNA126 by approximately 78.9% (p<0.05 vs. control group), but the virus infection had no effect on the expression of intracellular miRNA126 (Fig 4A). The decrease in miRNA126 significantly enhanced intracellular BCL-2 gene mRNA levels (P<0.05 vs. control group, Fig 4B). Moreover, knock-down of VEGF eliminated the regulatory effect of miRNA126 on BCL-2 (Fig 4B). To demonstrate that VEGF regulates BCL-2 expression by activating the transcription factor AP1, we constructed BCL-2 recombinant viruses with BCL-2 promoter and the CMV promoter, and analyzed whether the regulation of BCL-2 is mediated by AP1 by observing BCL-2 gene transcription efficiency post AP1 gene intervention. The data showed that BCL-2 recombinant viruses carrying either promoter all expressed high levels of BCL-2 in 293 cells. Overexpression of VEGF further enhanced BCL-2 expression under the control of its own promoter, while the BCL-2 levels expressed under the control of the CMV promoter gene remained unchanged (Fig 4C). In addition, the enhancement of BCL-2 expression under its own promoter by VEGF overexpression was significantly attenuated by AP1 gene silencing (p<0.05 vs. the group without AP intervention) (Fig 4D). Thus, the regulation of BCL-2 by VEGF is largely mediated by AP1.

### Effects of Targeted Expression of miRNA126 on Apoptosis of RAECs and ARECs and Related Proteins

**Fig 5:**
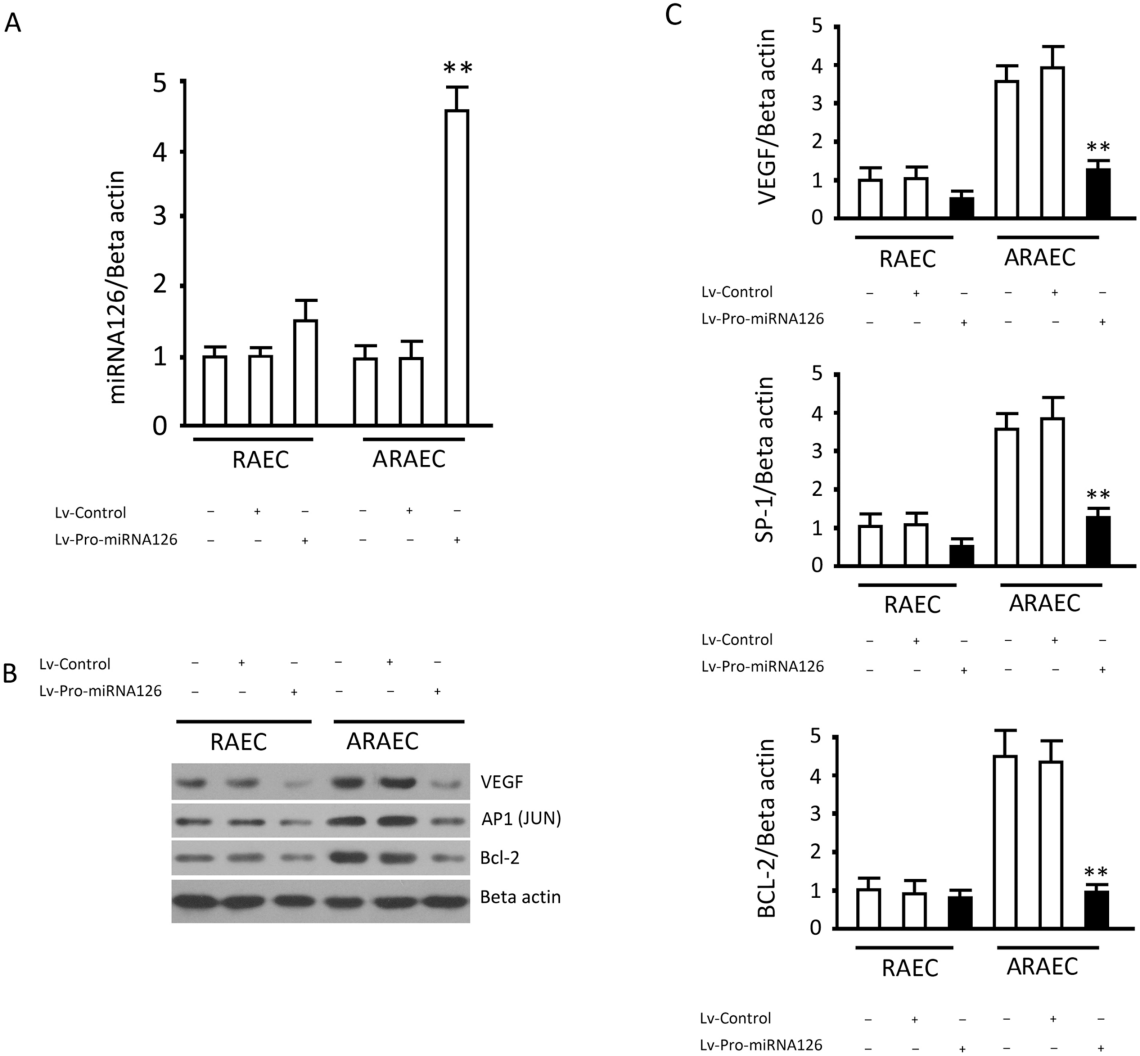
Effects of targeted expression of miRNA126 on apoptosis-related proteins in RAECs and ARECs. (A, B, and C) RAECs and ARAECs were infected with Lv-control or Lv-Pro-miRNA126 and BCL-2, AP1 (JUN), VEGF, SP1 and BCL-2 proteins were detected. Results are the means ± SD of at least 3 separate experiments. **, p<0.01, when compared to the corresponding Lv-control group.

The miRNA expression vectors carrying the BCL-2 promoter effectively expressed mature miRNA126 in endothelial cells (miRNA126 increasedby a factor of 4.2 in AREACs or 1.5 in REACs, Fig 5A). These results were also independently verified by protein detection. A miRNA126 expression vector carrying the BCL-2 promoter inhibited VEGF expression in apoptosis-resistant endothelial cells and ultimately influenced the expression of BCL-2 by the AP1 transcription factor (Fig 5B and 5C). These results suggested the miRNA control of the BCL-2 promoter was more effective to facilitate miRNA126 transcription in cells with high expression of BCL-2.

### Time-course of Targeted Inhibition by Lv-Pro-miRNA126

**Fig 6:**
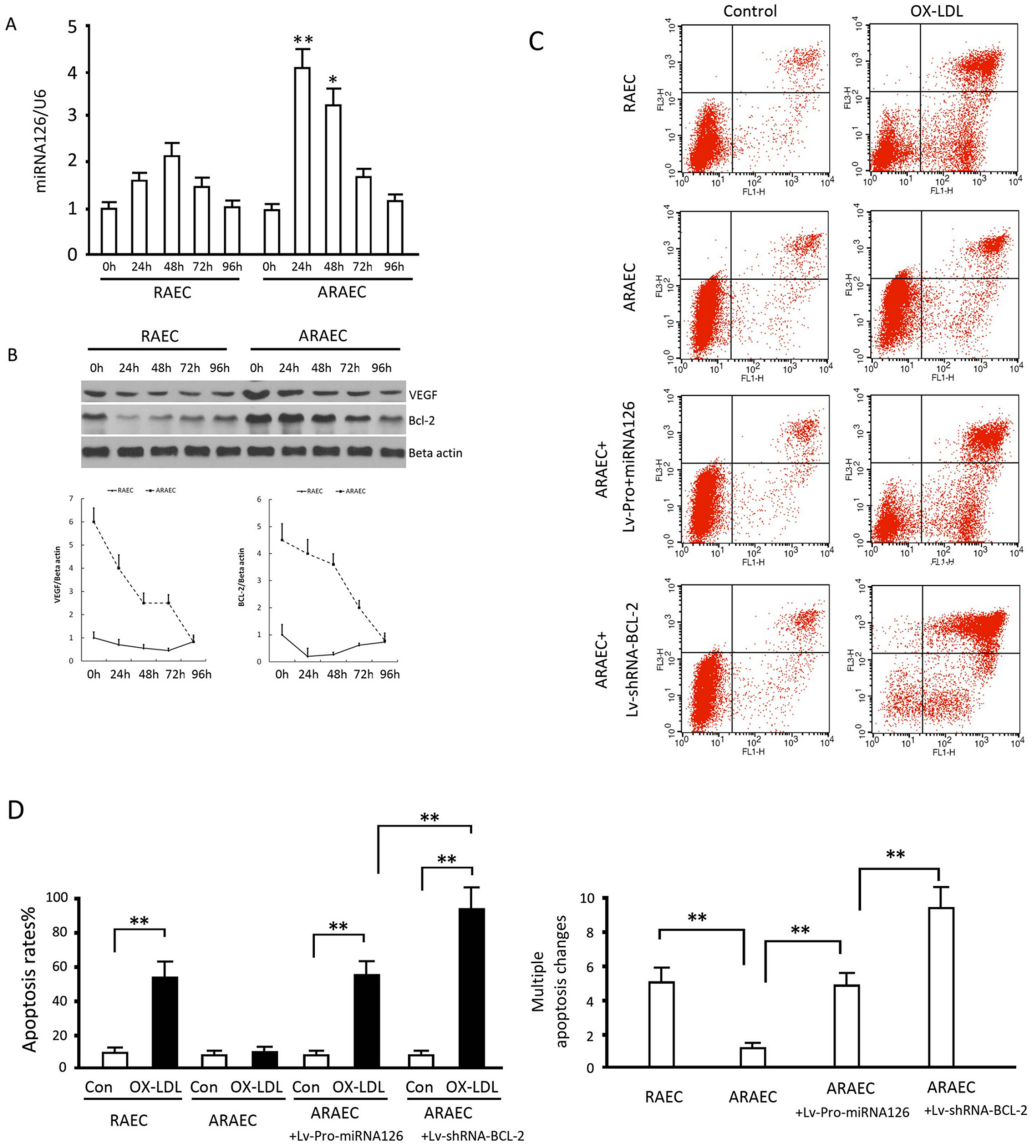
Detection of Lv-Pro-miRNA126 expression over time. (A, B) RAECs and ARAECs were infected with Lv-Pro-miRNA126, cultured for indicated times and miRNA126 RNA, VEGF and BCL-2 protein levels were assessed by quantitative PCR and western blotting, respectively. (C, D) Uninfected RAECs and ARAECs or ARAECs infected with Lv-Pro-miRNA126 or Lv-shRNA-BCL-2 were treated with or without OX-LDL for 48 hours and subject to an apoptotic assay. Representative plots are shown. Data are the means ± SD of at least 3 separate experiments. **, p<0.01, when compared to the RAECs group or the indicated group.

In cells overexpressing BCL-2, the transcriptional activity of the BCL-2 promoter is regulated by the overexpression of VEGF, and miRNA126 overexpression might inhibit BCL-2 transcription via AP1. Thus, we would expect that there would be a cyclic process, which may lead to flexible regulation. To test this hypothesis, we detected the time-course of miRNA126, VEGF and BCL-2 expression. The results showed that miRNA126 transcription guided by the Bcl-2 promoter was significantly increased at 24 and 48 hours in apoptosis-resistant endothelial cells while which was similar throughout the observation period in the normal endothelial cells (Fig 6A). In normal endothelial cells, VEGF and BCL-2 levels remained almost constant at low level (returned to baseline after small degree decrease). In AREACs, protein expression of both VEGF and BCL-2 declined continuously (Fig 6B). At 96 hours after infection, VEGF and BCL-2 protein levels were identical between the two groups. We infected ARAECs with Lv-pro-miRNA126 or Lv-shRNA-BCL-2 separately and examined their effects on apoptosis. We found that 96 hours after infection with Lv-Pro-miRNA126, the OX-LDL-induced apoptosis ratio was nearly the same in the apoptosis-resistant endothelial cells as that in normal endothelial cells, while BCL-2 gene silencing increased the apoptotic rate to approximately 94.2% in apoptosis-resistant endothelial cells (Fig 6C and 6D). These results suggested there was a flexible regulation or a negative feedback mechanism when the BCL-2 promoter was used to guide the expression of miRNA126 in BCL-2 overexpression cells. Thus, high BCL-2 expression could resulte in high expression of miRNA126, which in turn inhibited VEGF expression and BCL-2 expression. The decreased efficiency of the BCL-2 promoter also reduced the expression of miRNA126, making the levels infinitely close to that in normal endothelial cells.

### Effects of miRNA126 on Inflammatory cytokines and the MAPK Pathway

**Fig 7:**
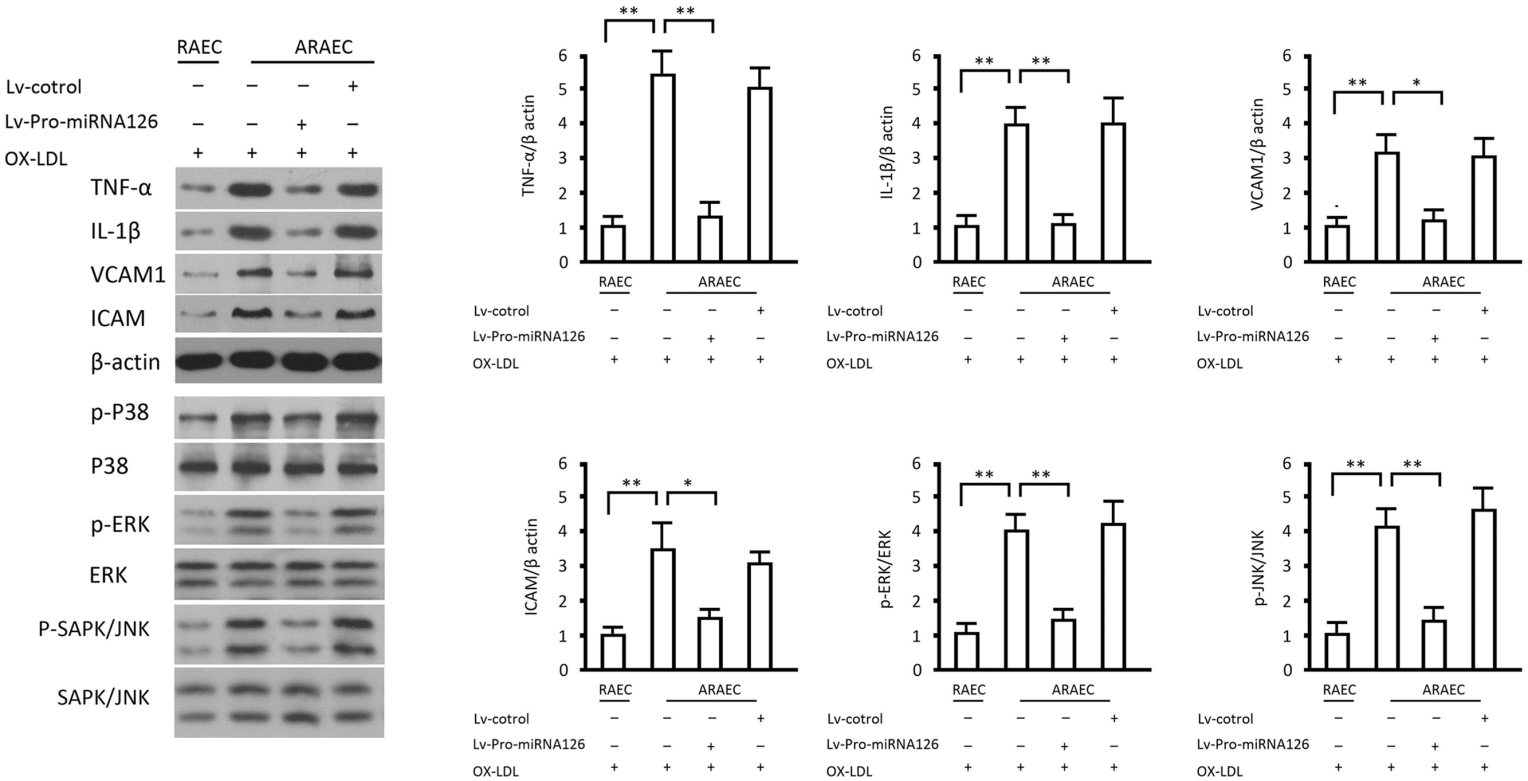
Effects of miRNA126 on inflammation-related factors and the MAPK pathway in apoptosis-resistant endothelial cells. Uninfected RAECs and ARAECs or ARAECs infected with Lv-Pro-miRNA126 or Lv-shRNA-BCL-2 were treated with or without OX-LDL for 48 hours and MAPK pathway related proteins and inflammation-related factors were detected by western blotting. Representative immunoblots and data representing the means ± SD of at least 3 separate experiments are shown. **, p<0.01, when compared to the indicated group.

OX-LDL increased expression of inflammatory cytokines and activated the MAPK pathway more effectively in apoptosis-resistant endothelial cells than in normal endothelial cells. Compared with the normal endothelial cells stimulated by OX-LDL, apoptosis-resistant endothelial cells treated with 50 μg/mL of OX-LDL expressed significantly higher levels of TNF-α, IL-1β, VCAM1 and ICAM (p<0.05), these effects could be significantly hampered by overexpression of miRNA126 (p<0.05 vs. the group without miRNA126 intervention) (Fig 7). The results on the MAPK pathway coincided exactly with the results of inflammatory factor assays (Fig 7). These data indicate that the inflammatory reaction in apoptosis-resistant endothelial cells post ox-LDL was stronger partly due to suppressed miRNA126 expression. Moreover, miRNA126 overexpression inhibited activation of the MAPK pathway by inhibiting VEGF. Attenuated MAPK pathway activation not only decreased the production of inflammatory cytokines, but also inhibited the formation of AP1 by Fos and jun, which consequently reduced the BCL-2 levels in apoptosis-resistant endothelial cells and, eventually, decreased their resistance to apoptosis.

## Discussion

The major findings of present study are as follows: 1. There is a negative correlation between expressions of miRNA126 and VEGF and BCL-2 in ox-LDL induced apoptosis-resistant endothelial cells. 2. Conditional miRNA126 overexpression could downregulate VEGF and BCL-2 expression and reduce the resistance to apoptosis in apoptosis-resistant endothelial cells and these effects are partly mediated through AP1. Moreover, miRNA126 overexpression inhibited activation of the MAPK pathway by inhibiting VEGF. Attenuated MAPK pathway activation might be responsible for reduced production of inflammatory cytokines and downregulated AP1 via Fos and jun, which may consequently reduce the BCL-2 levels in apoptosis-resistant endothelial cells and, eventually, decreased their resistance to apoptosis.

It has been established that activation of endothelial cells plays an important role in the development of atherosclerosis [15, 16]. Some studies showed that excessive proliferation of endothelial cells contributed to the formation of plaques and plaque vulnerability. Therefore, searching for factors and related mechanisms, which are involved in activating endothelial cells, is becoming a focus of current study related to the pathogenesis of atherosclerosis and control of vulnerable plaques. Apoptosis-resistant endothelial cells research is mainly active in the field of pulmonary arterial hypertension. Promoting apoptosis of pulmonary vascular endothelial cells is a useful strategy for reducing pulmonary artery pressure and right ventricular hypertrophy [17]. In the repair process of a vascular injury, the endothelial cells of new blood vessels express high levels of VEGF, while normal endothelial cells do not express VEGF and KDR [18]. Levy et al. found that intimal proliferation exists in the walls of pulmonary arterioles in patients with irreversible pulmonary hypertension, which is associated with impaired endothelial cell apoptosis due to the expression of an anti-apoptotic protein, BCL-2 [19]. In the present study, we established apoptosis-resistant endothelial cells from rat aortic endothelial cells stimulating by OX-LDL. We found that these cells had a stronger proliferative activity compared to normal endothelial cells. Moreover, compared with normal endothelial cells, VEGF and BCL-2 protein levels were significantly increased, making them a labile factor of atherosclerotic plaques [20].

Currently, miRNA research has achieved many breakthroughs in cardiovascular development and diseases [21]. Zhao et al. established a cardiac specific miR-1 transgenic mouse model and found that up-regulated expression of miR-1 leads to limited differentiation and development of cardiac ventricles [22–24]. Through miRNA expression profile analysis in human umbilical vein endothelial cells, Poliseno et al. identified 27 miRNAs expressed at high levels in HUVECs and their potential target genes include Flt-1, Flk-1, c-kit, Tie-2 and other important angiogenic factor receptors. This group also found that c-kit, a gene playing an important role in endothelial cell differentiation and angiogenesis, is a target of miR126—a microRNA cluster highly expressed in vascular endothelial cells [25]. Further studies confirmed that miR126 affects the migration of endothelial cells and tube formation. Through profile screening, we found that miRNA126 expression differed greatly in normal endothelial cells and in endothelial cells adjacent to atherosclerotic plaques. We also found that miRNA126 expression in endothelial cells adjacent to lesions was significantly lower than that in endothelial cells. We also identified VEGF as one of its target genes and confirmed that miRNA126 could negatively regulate VEGF in endothelial cells. our results showed that miRNA126 expression was significantly downregulated in apoptosis-resistant vascular endothelial cells compared to normal endothelial cells. Taking into consideration the high expression of miRNA126 in normal vascular endothelial cells and low expression in apoptosis-resistant endothelial cells, we speculated that upregulating miRNA126 expression might be a promising strategy to stabilize endothelial system function and vulnerable plaques through downregulating the expression of VEGF and BCL-2. We knocked down the expression of the BCL-2 gene in apoptosis-resistant endothelial cells and found that the apoptosis resistance decreased gradually with lower BCL-2 expression, indicating there is a definite link between BCL-2 expression and apoptosis resistance.

BCL-2 family members play crucial roles in apoptosis [26, 27, 28]. BCL-2 is a negative regulator of cell death and protects cells from apoptosis caused by external stimuli. The protein is mainly located in cytoplasmic face of the outer mitochondrial membrane, endoplasmic reticulum (ER), and nuclear envelope. It has membrane binding activity, which is extremely important to its function. Experimental results showed that the loss of membrane localization weakened its anti-apoptosis activity [29]. BCL-2 in mitochondrial membranes inhibited apoptosis at least three levels: (1) BCL-2 changes the mitochondrial thiol redox state to control the membrane potential, (2) BCL-2 regulates the permeability of mitochondrial membrane for a number of apoptotic protein precursors, and (3) BCL-2 anchors the pre-apoptotic protein Apaf-1 and other proteins to the mitochondrial membrane, which inhibits their apoptotic functions. Studies on BCL-2, including its functions and its transcriptional regulation, is one of the most popular topics in the field of apoptosis [30]. Various growth factors and cytokines can induce the expression of BCL-2, however, the responses to different stimuli varied in different cells and the regulation of BCL-2 is diverse and complex. The AP1 transcription factor usually exists as a homo- or heterodimer composed of family members of jun (c-jun, junB, junD) and Fos (Fra-1, Fra-2, c-fos, fosB) and binds to 5'-GTGAGCTCAG-3’. In this study, we performed bioinformatic analysis to identify an AP1 binding site approximately 473 bp before the starting codon of BCL-2 gene and proved that VEGF regulates BCL-2 via AP1. We also found that VEGF can activate the MAPK pathway, which in turn phosphorylates AP1 and enhances its transcription factor activity.

So far, we confirmed the regulational relationship between miR126, VEGF and BCL-2. To avoid affecting expression of VEGF in normal endothelial cells, we used the specific BCL-2 promoter in a miRNA126 overexpression system. Before these experiments, we did not have a sense on how the miRNA126 overexpression induced by the BCL-2 promoter might affect AP1 activity. The experimental data showed that the negative feedback of this cycle produced interpretable results. miRNA126 downstream of the BCL-2 promoter was only expressed in cells that had high levels of BCL-2 expression, which in turn reduced the transcriptional activity of the BCL-2 promoter and reduced transcription of miRNA126, thus, achieved a balance. The balance between BCL-2, miRNA126 and VEGF was very close to that of normal endothelial cells. The reason we did not silence VEGF directly was that the targeted promoter was not suitable for shRNA transcription. The reason we did not use direct regulation of AP1 or BCL-2 via miRNAs was that miRNA126 can moderately change a variety of adverse factors.

In the development of atherosclerosis, the damage of endothelial cells results in EMT of endothelial cells, an important process of vasculopathy. Interestingly, we found that the secretion of miRNA126 from ARAECs was reduced, resulting a lower level of local miRNA126 in serum, and possibly reducing miRNA126 entering the endothelial cells from the serum; therefore, reduction of miRNA126 in endothelial cells certainly leads to the change in its target protein, VEGF. It is worth further research whether this is the direct cause of EMT of endothelial cells [31].

In summary, we constructed a miRNA126 expression vector containing the BCL-2 promoter and specifically expressed miRNA126 in endothelial cells with high BCL-2 expression levels and, consequently, this strategy reduced VEGF and BCL-2 levels to nearly normal levels in apoptosis-resistant endothelial cells. The regulation mechanism of feedback control did not affect the expression of VEGF in normal endothelial cells, but inhibited VEGF and BCL-2 expression in apoptosis-resistant endothelial cells. This is important because high levels of BCL-2 expression are essential to the atherosclerosis repair process orchestrated by endothelial cell activation. The inhibition of VEGF by miRNA126 impaired activation of the MAPK pathway, and resulted in two actions: (1) Inhibition of the MAPK pathway directly reduced the expression of many inflammation-related cytokines responsible for the increased instability of atherosclerosis plaques [32, 33, 34] and (2) Inhibition of the MAPK pathway inhibited formation of FOS and JUN dimers, which decreased the activity of AP1, down-regulated BCL-2, and which might be able to reduce the apoptosis-resistant endothelial cells at lesion area. Fture in vivo studies are warranted to verify if conditional miRNA126 overexpression might regulate the excessive activation of the endothelial system and reduce the generation of apoptosis-resistant endothelial cells in the atherosclerotic plaques, and ultimately used to reduce plaque formation and plaque rupture in patients with atherosclerosis. It is to note that angiogenesis and anti-apoptosis are somehow wanted responses for patients with ischemic heart diseases, thus, the optimal strategy should be the localized miRNA126 overexpression in vascular atherosclerotic plaques.
